# May furtive predation provide enemy free space in ant-tended aphid colonies?

**DOI:** 10.1371/journal.pone.0204019

**Published:** 2018-10-10

**Authors:** Benoit Guénard, François Dumont, Bruno Fréchette, André Francoeur, Éric Lucas

**Affiliations:** 1 Département des Sciences Biologiques, Université du Québec à Montréal, Montréal, Qc, Canada; 2 University of Hong Kong, School of Biological Sciences, Hong Kong SAR, China; 3 Université du Québec à Chicoutimi, Chicoutimi, Qc, Canada; University of California San Diego, UNITED STATES

## Abstract

In furtive predation, a predator is able to exploit its prey without generating significant defensive behaviors from them. However, in aphidophagous guild, if furtive predator can benefit from dilution effects generated by the aphids, they also suffer from intraguild predation from more mobile and active-searching predators. In this context ant-tended aphid colonies might not only represent an important food source but also potentially an enemy-free space for furtive predators if they remain unharmed by ants while other active predators are being repelled. Here we use the furtive predator *Aphidoletes aphidimyza* and two distinct instars of an active-searching predator, the Asian ladybeetle *Harmonia axyridis*, to test hypotheses related to predator persistence within aphid colonies in presence of ants. Our results show that persistence rate over time of the furtive predator was not affected by ant presence while it was strongly reduced for both instars of the active-searching predator. Furthermore, when ran in paired trials within ant-tended aphid colonies, furtive predator persistence rate was significantly higher than for active-searching predators, with these latter always leaving the plants quicker. Finally, we tested the importance of predator mobility in detection susceptibility and aggressive responses in ants using mobile and immobile active-searching predators. While the number of antennal palpations was similar for both treatments indicating similar detection rate, the number of ant attacks was significantly higher on mobile individuals highlighting the importance of movement in triggering aggressive responses in ants. Overall our results indicate that furtive predation represents an efficient strategy to limit ant aggressions, while the exclusion of active-searching predators might create an enemy-free space for furtive predators within ant-tended aphid colonies.

## Introduction

Predation represents a major cost to herbivorous organisms either directly through consumption or indirectly through modified foraging patterns and physiology, which ultimately affect reproductive success [1]. Some herbivores engage in protection mutualisms to create enemy-free spaces [2–3]and avoid costs incurred by predation. In such associations, the protected herbivore supplies food (e.g. honeydew, nutritive glandular secretions) to an organism that, in turn, provides defense against natural enemies. The protection mutualism between honeydew-producing insects and ants has been extensively described as a canonical example of food-for-protection mutualisms [4–8]. These insects supply ants with honeydew, which is an easily accessible resource that is spatially and temporally predictable [9]. In return, ants guard herbivores against pathogens (Buckley 1987), predators, and/or parasitoids [4, 10–12].

Although these food-for-protection mutualisms are beneficial to both parties, the defensive services provided by ants are not perfect. Species participating in these mutualistic interactions occasionally coexist with exploiters—non-mutualist species that have evolved adaptations to take advantage of this mutualistic relationship without providing benefits to the initial partners [13–15]. For instance, some natural enemies are able to exploit ant-tended honeydew producers without being attacked by ants [16]these aphid enemies simply avoid contact with ants, either spatially or temporally [17–18]Other natural enemies remain in direct contact with ants but do not trigger an attack response due to morphological, physiological, behavioral and/or chemical adaptations. The aphid parasitoid *Lysiphlebus cardui* (Marshall) (Hymenoptera: Braconidae) uses cryptic movements [19]and chemical mimicry [20] to avoid attacks from ants in proximity to a defended aphid colony, while the lacewing *Chrysopa slossonae* (Banks) (Neuroptera: Chrysopinae) can exploit ant-tended aphid colonies using camouflage [21].

In the latter case, these natural enemies may also benefit from an *enemy-free space* within ant-tended colonies and use the defensive behavior of ants to their own advantage. Furtive predators (i.e. predators that live amongst their prey without triggering defensive responses) known to benefit from aphid group defense mechanisms [22–24] could for instance also benefit from the ant presence. For instance, the furtive aphidophagous predator, *Aphidoletes aphidimyza* (Rondani) (Diptera: Cecidomyiidae), often share its habitat with other active-searching aphid predators [25–27].Since *A*. *aphidimyza* eggs and larvae are highly susceptible to intraguild predation (IGP) [28–31] living in a high density aphid colony is a way to reduce the risk of being preyed upon. Lucas & Brodeur [22] showed that the size of an aphid colony has an effect on the survival of the furtive predator in presence of lacewing larvae. Moreover, the furtive predator *A*. *aphidimyza* preferentially occupied the center of aphid colony, which increased survivorship in presence of IGP predators [24]. *Aphidoletes aphidimyza* therefore benefits not only from collocation with a food resource, but also from a dilution and a selfish herd effect which may provide protection from IGP. However, the benefit of any dilution effect will depend on the rate of disturbance generated by the intraguild predator. Lacewing larvae tend to disorganize aphid colonies during predation and do not benefit from dilution or selfish herd effects [22, 24].Previous studies have shown that movement is an important part of prey identification in ants [4, 32–33] and predators with slow movements or camouflage behaviours may avoid being attacked by ants [4, 34]. The slow motion of *A*. *aphidimyza* larvae, combined with their ability to occasionally cover themselves with aphid exuviae, dead or paralyzed aphids [22] could further improve their camouflage and avoid detection by ants.

In this study, we tested the ability of the furtive predator, *A*. *aphidimyza*, to avoid detection and attacks by ants and thus present higher persistence rate within aphid colonies than small or large active-searching predators. Second, we tested the hypothesis that slow movement is the key factor to prevent detection from ants. We predicted that immobilised active searching predators would be less detected and attacked by ants than mobile ones.

## Methods

### Organisms

One *Lasius niger* (L.) (Hymenoptera: Formicidae) colony of 5,000 individuals, including a gyne and brood, was collected during the summer of 2004 and was brought to the laboratory. The colony was housed in a plastic box connected to other boxes by plastic tubes of 2 cm in diameter and maintained at a constant temperature between 22–24°C. To allow manipulations, the upper parts of the boxes were opened with insect barrier strips (Tanglefoot) applied inside the boxes to prevent ant escapes. A foraging area, accessible to the ants and able to contain 12 plants was set to perform the experiment. Before each experiment, the ant colony was deprived of food for 48h to ensure ants need for sugars and an efficient response in tending aphid colonies of *Aphis fabae* (Scopoli) (Hemiptera: Aphididae) reared on broad bean plants (*Vicia faba* L.). This species is a facultative myrmecophilous species with well-known mutualistic interactions with *L*. *niger* [10, 35].

Three types of predator were used in these experiments: a furtive predator *A*. *aphidimyza* (late instar vermiform larvae; body size approximately3mm long), a small active-searching predator *Harmonia axyridis* (Pallas) (Coleoptera: Coccinellidae) (2^nd^ instar campodeiform larvae; body size approximately3.5mm), and a large active-searching predator *H*. *axyridis* (4^th^ instar campodeiform larvae; body size 7.7–10.7 mm) [36].

### Persistence of each type of predators in the presence and the absence of ants

Broad bean plants (30 cm tall) hosting 300–500 *A*. *fabae* were selected for the experiments. A single predator from one of the three types (furtive, small active, large active) was introduced on each plant at the vicinity of the aphid colony. Thirty minutes after the introduction of the predators, 25 plants were placed in contact with ants while 25 others served as control (without ants); with six to eight plants introduced simultaneously in the foraging area of the ants’ colony per experimental run. Plants were physically separated from one another without contact for the entire duration of the experiment. For each trial, the start of the experiment began once an ant discovered the aphid colony.

Presence or absence of predators on plants was determined after 24h. After 24h, plants were carefully inspected and dissected.

### Persistence of furtive vs active-searching predators on paired plants in the presence of ants

In this experiment we aimed at comparing the persistence of furtive and active-searching predators in presence of ants.

Plants were paired according to their size (± 5 cm), their total leaf area, and the size of the aphid colonies they hosted (± 50 individuals). To obtain similar groups of plants, leaf area and/or aphid population size have been modified. Aphids on the lower part of the plant were removed to obtain colonies of 300–500 aphid individuals per plant.

One predator, either furtive (*A*. *aphidimyza)* or active-searching (*H*. *axyridis*), was then introduced on the upper part of the plant. Two different combinations of predators were tested:

- Furtive Predator (FP) paired with small Active-searching Predator (SAP).
- Furtive Predator (FP) paired with large Active-searching Predator (LAP).

To control for predator disturbance, plants were placed in the ants’ foraging area thirty (30) minutes after the predator introduction; with a maximum of 6 plants hosting a furtive predator and 6 plants hosting an active-searching predator (Small (n = 18) or Large (n = 17), depending on the combination tested). As for the previous experiment, the first discovery by an ant with the aphid colony determined the beginning of the test; with presence or absence of predators recorded after 1h, 2h, and 24h. The proportions of each predator type (FP, SAP and LAP) present on the plants at the end of each time period (i.e., persistence rates) were calculated and compared in a contingency table. Subsequently, similar analyses were performed to compare these results with those of the first experiment.

To determine whether ant attacks were directed preferentially towards a type of predator, the presences of predators were observed for each paired plants after each period; with the three following scenarios possible for any given period: (1) one of the two predators was missing, (2) both predators were missing, and (3) both predators were present.

### Impact of mobility on predator persistence in the presence of ants

In this experiment the hypothesis that the higher persistence of furtive predators is due to their slower movement or immobility, thus enabling them to remain undetected by ants was tested.

The experimental design was similar to the previous experiment, with the large active-searching predator *H*. *axyridis* 4^th^ instar larvae used, either alive (mobile) or previously killed by frost (immobile). Mobile larvae were introduced on plants thirty (30) min prior to the introduction of plants in the ants' foraging area. Immediately after plants were introduced in the foraging area, immobile larvae (kept at room temperature for at least one hour prior the experiment) were gently placed on the upper surface of a leaf, 2 cm away from the aphid colony. Trials started with the first attendance of aphid colonies by ants. Twenty three replications were performed.

The presence or absence of each type of predator was observed after 1, 2, 3, 4, and 24h. Furthermore, the behaviour of ants towards *H*. *axyridis* larvae was observed during the first hour of the test. Ant behaviours were classified into one of two categories: (1) *Antennal palpation*, i.e., exploratory behaviour, or (2) *Aggression* (light nibbles, haul of the predator, attack with mandibles, or spit of formic acid); which together could lead to three possible sequences: (1) *Antennal palpation without attack*, (2) *Direct attack*, or (3) *Antennal palpation followed by attack*.

In all tests, blinded methods were use when behavioral data were recorded and analyzed to minimize observer bias.

### Statistical analysis

The persistence of each type of predator after 24 hours in presence or absence of ants was analyzed with a generalized linear model (GLM) for binomial data.

The persistence of furtive and active-searching predators in the presence of ants was tested with a generalized linear mixed model (GLMM) for binomial data. The type of predator (furtive or mobile) and the time was included in the model as well as the interaction between these fixed factors. The plant ID was included in the model as a random effect to control for repeated measures. Experiments on small (SAP) and large (LAP) active-searching predator was tested independently and analysed as such.

The same approach was used to test the impact of mobility on predator persistence in the presence of ants.

The different types of behaviour expressed by ants were compared with a contingency table for the different periods. Paired t-tests compared which of the predator was excluded first.

All analysis were run on R [37].

## Results

In all trials, ant attendance of aphid colonies began during the first hour following plants' introduction, and was quickly followed by the recruitment of additional ants.

### Persistence of each type of predators in the presence and the absence of ants

**Fig 1:**
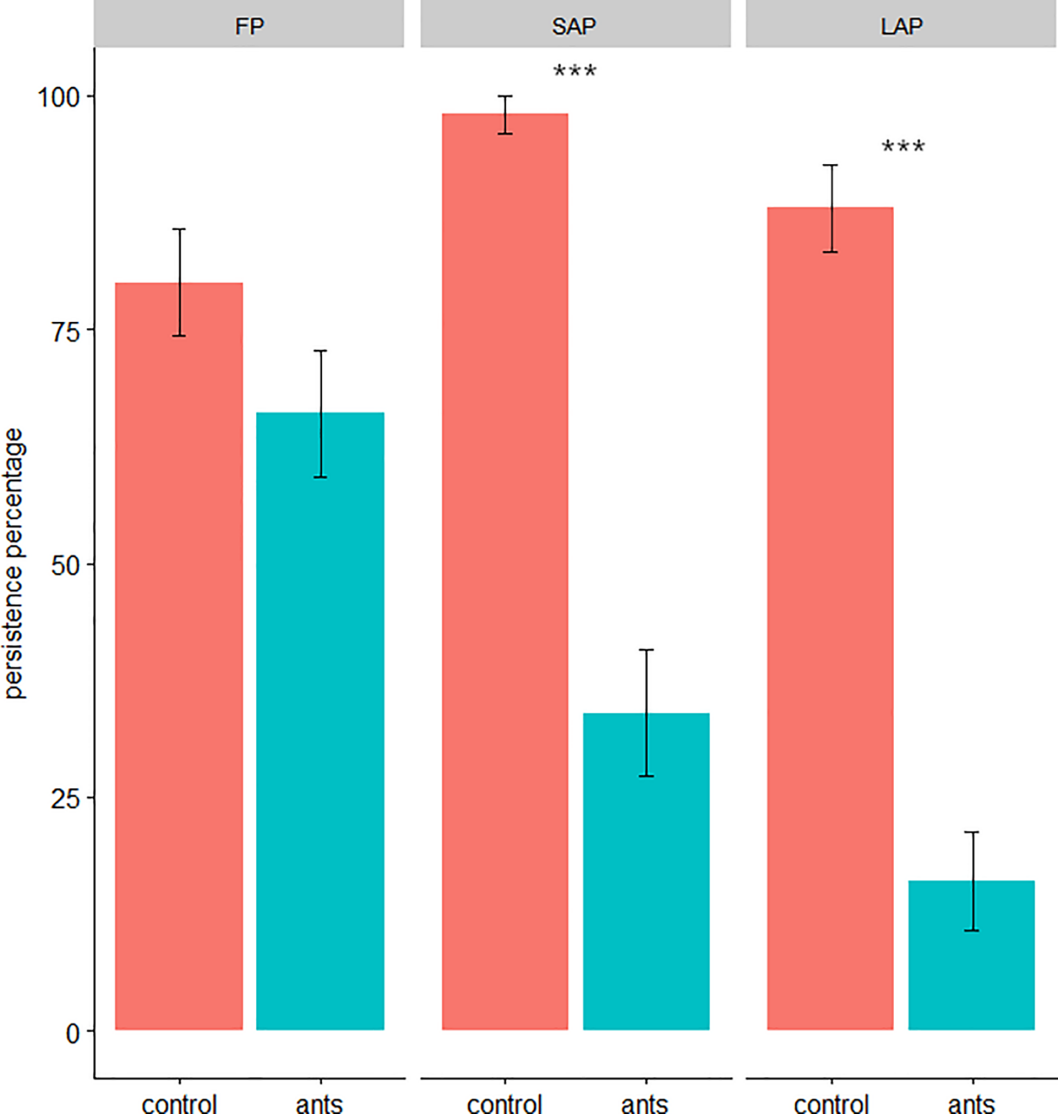
Persistence of the different predators after 24h in presence or not of ants. FP = furtive predator (*A*. *aphidimyza*), SAP = small active-searching predator (second instar of *H*. *axyridis*) and LAP = large active-searching predator (fourth instar of *H*. *axyridis*).

Ants had no significant impact on the persistence of the larvae of the FP (*A*. *aphidimyza* larvae) after 24 h (β = -0.99 ± 0.59, z = -1.69, p = 0.09) (Fig 1). In contrast, ants had a significant negative impact on the SAP (*H*. *axyridis* 2^nd^ instar) after 24h (β = -25.39 ± 7.12, z = -3.57, p = 0.0004), and on the LAP (*H*. *axyridis* 4^th^ instar) after 24h (β = -24.64 ± 5.95, z = -4.14, p < 0.0001).

### Persistence of furtive vs active-searching predators in the presence of ants

#### Furtive predator (FP) paired with small active-searching predator (SAP)

**Fig 2:**
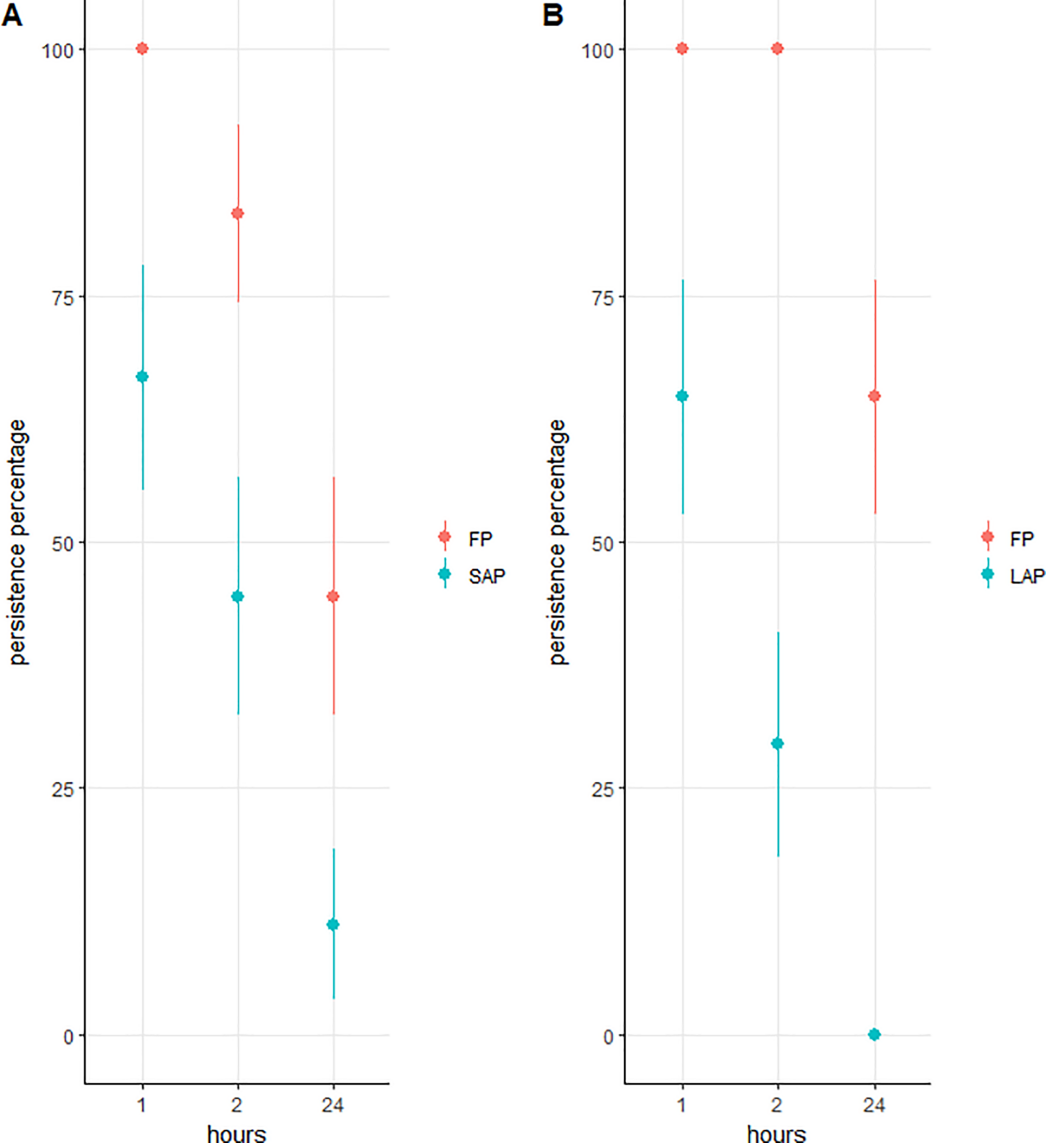
Persistence of the different predators through time on ant-attended colonies (A) Comparison of furtive predator (FP) (*A*. *aphidimyza*) and small active-searching predator (SAP) (second instar of *H*. *axyridis*) persistence. B) Comparison of furtive predator (FP) (*A*. *aphidimyza*) and large active-searching predator (LAP) (fourth instar of *H*. *axyridis*) persistence.

The persistence of both the FP and SAP predator decreased over a period of 24h (β = -0.22 ± 0.09, z = -2.52, p = 0.01). On average the FP had a higher persistence rate than SAP (β = -3.90 ± 1.82, z = -2.15, p = 0.03) (Fig 2A; Table 1). Significant differences were observed rapidly, with 100% and 67% of persistence after 1h respectively for the FP and SAP, 83% and 44% after 2h and 50% and 11% after 24h. No interaction between the species of predator and the time was observed (β = 0.01 ± 0.08, z = 0.13, p = 0.90) indicating that the persistence of both predator decrease at similar rate and that the difference in persistence between the two species is due to the rapid removal of the SAP.

**Table 1:** Persistence percentage of the furtive predator (*A*. *aphidimyza*), small active-searching predator (second instar larvae of *H*. *axyridis*) and large active-searching predator (fourth instar larvae of *H*. *axyridis*) after 2 h and 24 h in presence and absence of ants on aphid colonies of *A*. *fabae*.

	Persistence % after 2h			Persistence % after 24h		
Predators' type	With ants	Without ants	*P* Value	With ants	Without ants	*P* Value
Furtive predator	92 ± 5.5	88 ± 6.6	0.637	72 ± 9.2	48 ± 10.2	0.083
Small active predator	48 ± 10.2	100	**< 0.001**	20 ± 8.2	96 ± 4.0	**< 0.001**
Large active predator	24 ± 8.0	96 ± 4.0	**< 0.001**	8 ± 5.5	80 ± 8.2	**< 0.001**

#### Furtive predator (FP) paired with large active-searching predator (LAP)

The rate of persistence of the LAP predators decreased rapidly reaching 65% and 24% after, respectively, 1h and 2h (Fig 2B). No LAP persisted over a period of 24h. In contrast, all FP persisted during the first 2h when paired with LAP and 65% were still present on the plant after 24h. A significant interaction between the species and the time was thus observed (β = -17.54 ± 5.32, z = -3.30, p = 0.001) indicating that the ants first attacked the LAP over FP.

#### Impact of mobility on predator persistence in the presence of ants

**Fig 3:**
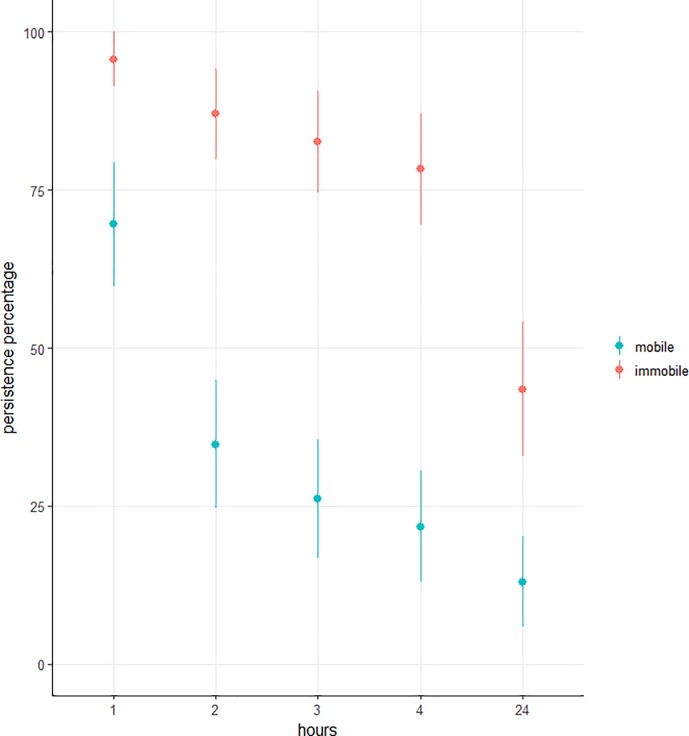
Persistence percentage through time on ant-attended aphid colonies of the mobile and immobile fourth larval instars of the active-searching predator *H*. *axyridis*.

Immobile predators persisted longer on plants than mobile predators (β = -6.48 ± 2.36, z = -2.74, p = 0.006). After 1h, 70% of mobile and 96% of immobile larvae were still present (Fig 3). After 2h, 35% of mobile and 87% of immobile larvae were still present, then respectively 26% and 87% after 3h, 22% and 82% after 4h, and finally 3% and 44% after 24h (Fig 3). Therefore, the persistence of both immobile and mobile predator decreased over time (β = -0.31 ± 0.12, z = -2.66, p = 0.008), but no interaction between mobility and time was observed (β = 0.07 ± 0.10, z = 0.68, p = 0.50).

**Fig 4:**
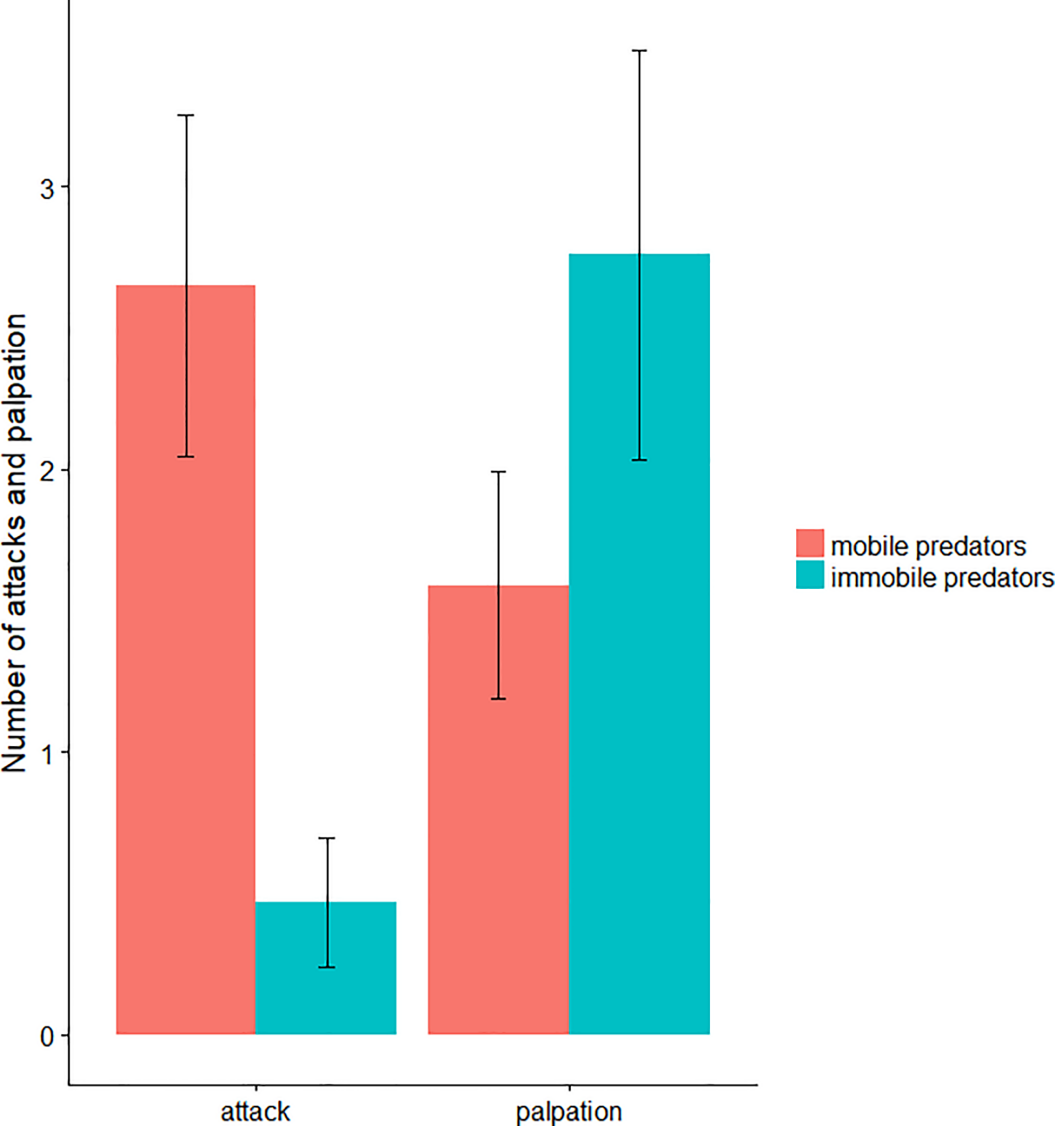
Number of attacks and palpations of ants on the mobile and immobile fourth larval instars of the active-searching predator *H*. *axyridis*.

Of 17 combinations observed, 27 and 47 antennal palpations were observed on mobile and immobile larvae respectively (χ^2^ = 2.8, *P* = 0.097). The average number of antennal palpations was 1.59 (SE = 0.40) on mobile larvae and 2.76 (SE = 0.73) on immobile larvae (χ^2^ = 1.055, *P* = 0.304). The mobile larvae were significantly more assaulted than the immobile larvae with a total of 45 and 8 aggressive behaviors observed on mobile and immobile larvae respectively (χ^2^ = 15.0, *P*<0.001) (Fig 4) representing an average of 2.65 (SE = 0.61) and 0.47 (SE = 0.23) aggressive behaviors per individual respectively (χ^2^ = 11.337, *P*<0.001).

**Fig 5:**
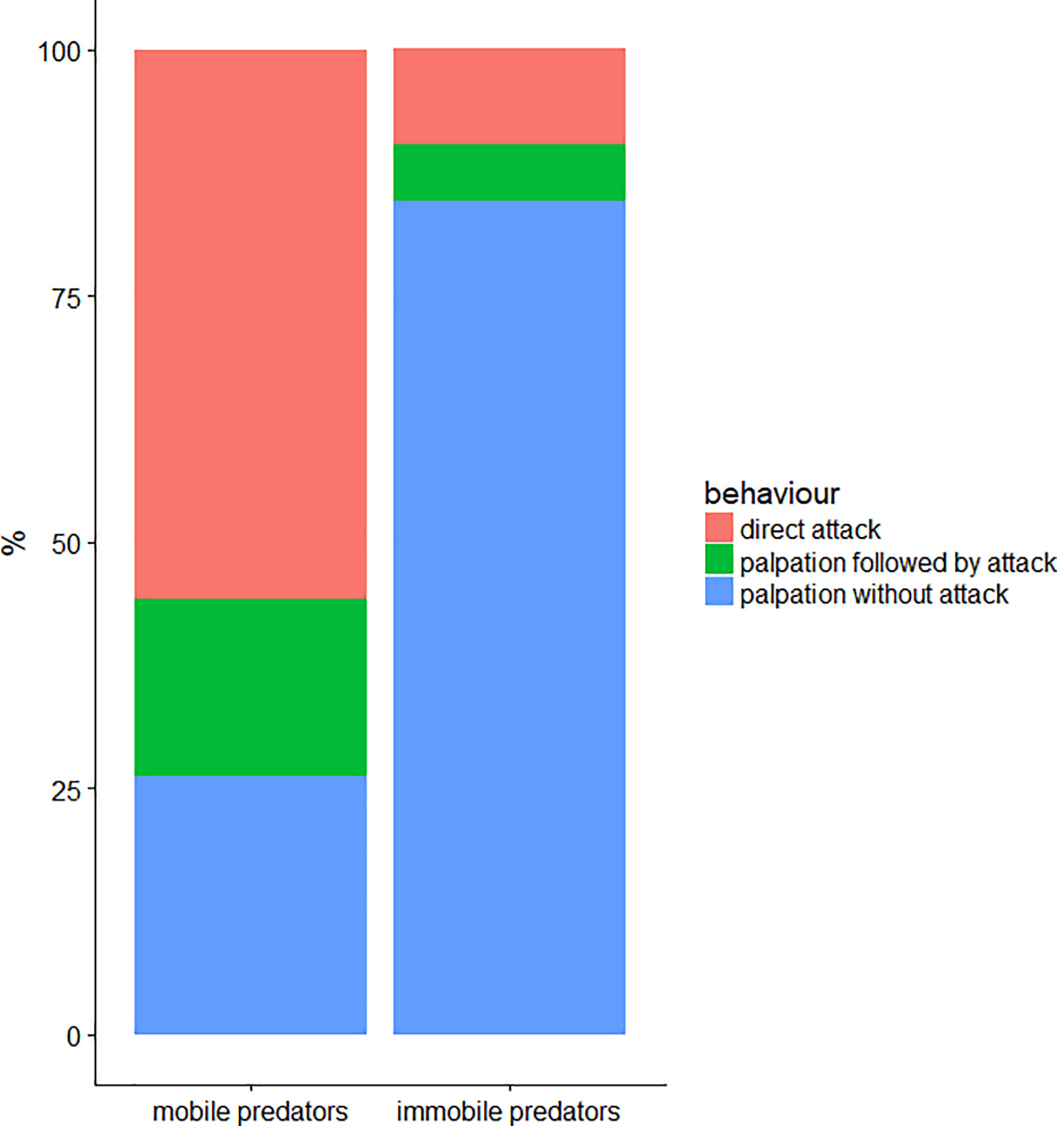
Percentage of the different observed behaviors of ants towards the mobile and immobile fourth larval instars of the active-searching predator *H*. *axyridis*.

There was a general significant difference between mobile and immobile larvae with regard to the relative proportion of behaviours expressed by ants (χ^2^ = 29.5, *P*<0.001) (Fig 5). Of the 17 replications, a higher number of direct attacks were observed on mobile than on immobile larvae, with an average of 2.00 (SE = 0.54) direct attacks per individual on mobile larvae and 0.29 (SE = 0.17) direct attacks on immobile larvae (Wilcoxon test, z = 3.3, *P*<0.001). The number of antennal palpations followed by attacks was also greater, with an average of 0.65 (SE = 0.17) on mobile larvae and 0.18 (SE = 0.10) on immobile larvae (Wilcoxon test, z = 2.2, *P* = 0.027). Regarding the number of antennal palpations without attack, a trend has been noted (Wilcoxon test, z = -1.9, *P* = 0.056): with averages of 2.59 (SE = 0.69) on immobile larvae and 0.94 (SE = 0.31) on mobile larvae.

## Discussion

The results of the experiments confirmed the hypothesis that furtive predators have a higher persistence than active-searching predators within aphid colonies tended by ants. The furtive predator *A*. *aphidimyza* was significantly less affected by ants than the two instars of larvae of the active predator *H*. *axyridis* which were commonly excluded from plants. Further, our results support the hypothesis that mobility might be one of the key-factors associated with ant detection of potential threat and aggressiveness; the mobile predators suffering higher aggression rates than non-mobile predators.

Several elements associated with furtive predation suggest that this behaviour could allow the persistence of *A*. *aphidimyza* larvae within ant-tended aphid colonies. For example, furtive predators do not cause significant defensive responses in aphids [22–23] and it was demonstrated that the alarm pheromones emitted by aphids warn ants of the presence of a potential predator [38]. Within our experiments, the furtive larvae of *A*. *aphidimyza* show similar persistence within aphid colonies in presence or absence of ants. While in both treatments, a decrease in the number of larvae over time was observed, particularly after 24 hours, this could be explained by the fact that older larvae drop from the plants in order to pupate in the soil [39]. Unlike the furtive predator, the active-searching predators were strongly affected by ants. To avoid the attacks of ants, it was observed that active larvae restricted their movements and remained away from the aphid colonies. Antennal palpations of ants on *H*. *axyridis* larvae sometimes caused the movement of legs or the escape of the larva: with these behaviours initiating attacks from ants. The escape of the active-searching predator occurred generally after several repeated attacks from ants, however it should be noted than whenever possible, ladybeetle larvae tried to avoid ants and to remain on the plant.

Which mechanisms may explain the differential susceptibility of furtive and active-searching predators? Our results support the hypothesis that slow movement is a major factor that enable furtive predator to avoid ant attacks. The low number of aggressive behaviours following antennal palpations on immobile ladybird larvae indicates that those are not identified as a potential enemy by ants. At the opposite, numerous attacks were observed following antennal palpation on mobile ladybird larvae, suggesting a possible aggressive response of ants to movement stimulations. The importance of movements for the detections of prey/intruders by ants is well known [40–42].However, other factors than mobility such as size, morphology, and chemical signature could potentially influence the detection of aphid natural enemies by ants. It should be noted that while the furtive *A*. *aphidimyza* larvae are slightly smaller than the smaller active-searching predator (coccinellid 2^nd^ instar larvae), ants responses to small and large active predators (2^nd^ and 4^th^ instars coccinellid) were similar. Thus, detection by ants does not seem to be significantly affected by size, suggesting this factor is negligible in our experiments. While the furtive *A*. *aphidimyza* larvae are vermiform, those of the active-searching ladybeetle are campodeiform, which may have influenced the response of ants. However, our results demonstrated that despite similar morphology, ants responded differently to mobile and immobile 4^th^ instar ladybird larvae. Thus, if morphology is an important factor, it is clearly not the main one responsible for ant aggressive responses. Finally, if a species chemical signature is a cue that triggers ant aggressive behaviour, we should expect equal number of attacks on mobile and immobile 4^th^ instar ladybird larvae; since immobile larvae were freshly killed, the chemical signatures of their cuticle should be similar to that of living (mobile) larvae. Furthermore, no reflex bleeding was observed in ladybird larvae during our experiments. Thus, the ladybird chemical signature was not responsible for triggering aggressive behaviour in ants. Therefore, our results suggest that if movement might not be the only factor triggering aggressive behaviour in ants, it appears in our experiment to be a major one.

One aspect not explored in this study is the possibility that the furtive predator might use chemical mimicry. The use of chemical mimicry is known in parasitoids [43–44], some ladybirds [45], and is suspected in myrmecophilous spiders[46] to prevent attacks from ants. This strategy may be subdivided in two categories, i.e. *ant colony mimicry* and *prey mimicry* [45]. For example, the parasitoid *Lysiphlebus cardui* (Marshall) (Hymenoptera: Braconidae) use chemical mimicry [47], possessing almost all host-specific compound of the aphid *A*. *fabae* [20]. Studies on the cuticular profile of the midge would be necessary to explore this factor and we thus cannot rule out this hypothesis.

Because of their limited mobility, the furtive *A*. *aphidimyza* larvae remain within the same aphid colony throughout their development [48]. Thus, at the time of oviposition, females determine the location of the eggs/larvae to come and therefore their likelihood of survival. It has been shown that females seek to increase the survival of their eggs/larvae by selecting specific sites depending on food availability, plant configuration or trichomes occurrence [29, 48]. A follow-up hypothesis would be to investigate whether or not *A*. *aphidimyza* females select preferentially oviposition sites where ant pheromones are present. *Aphidoletes aphidimyza* females lay eggs at night [39] when ants are usually less active (or inactive) and where their visual perception might be reduced, enabling them to avoid ant attacks. Such a preference was shown for some myrmecophilous Lycaenidae [49] and in the parasitoïd *L*. *cardui* [43]. However, empirical observations by Sentis et al. [50] indicated no relationship between ant-attendance and *A*. *aphidimyza* oviposition, which may be linked to a detrimental effect of ants on furtive predators.

According to their morphology, size and lack of defensive behaviours, the furtive predator *A*. *aphidimyza* seems much more vulnerable to attacks by ants than the larger and spiny active-searching ladybird predator. However, this study demonstrates that the furtive predator is actually the one who persists longer in ant-attended aphid colonies, while more active aphid predators also known to be intraguild predators of *A*. *aphidimyza* [22] were drove out from aphid colonies. As a result, ants through their patrolling activity and aggressiveness towards active predators might provide an enemy-free space for furtive predators.
